# Randomized Allocation to Treatment Groups and the Importance of Adjusting for Covariates

**DOI:** 10.6004/jadpro.2014.5.1.3

**Published:** 2014-01-01

**Authors:** Dustin Dickerson

**Affiliations:** From Billings Clinic, Billings, Montana

Research questions are concerned with changing a specific quantity by imposing an intervention or treatment. To find the answers to such questions, researchers must follow the scientific method to design a study that will gather quality evidence and lead to a logical conclusion. A common challenge to this process, whether testing a new drug or an educational intervention, is controlling for the effect of variables that may affect the response besides the one being studied. These variables are known as covariates. This article will discuss the importance of controlling for covariates and will describe statistical techniques to facilitate the process.

The ATLAS study (Davies et al., 2013), which is discussed in detail in the article by G. Lita Smith that begins on page 57, is a good example to consider here because it employs a clear and concise study question: "Is a 10-year tamoxifen treatment more effective than a 5-year treatment at reducing cancer recurrence and mortality in women with estrogen receptor (ER)-positive early breast cancer?" Additionally, the researchers use multiple strategies to minimize the effects of covariates, allowing for an objective comparison between treatment groups.

## Randomization and Generalizability

Statistics are a necessity to any research project due to the concept of generalizability. Not surprisingly, researchers want to apply their results to a broader group of people, not just the participants in the study. An accurate measure of the treatment effect—let alone generalizing findings from a sample to a larger population—requires that the research abide by certain rules. Two vital rules must be followed: The sample must be representative of the population of interest, and the treatment groups must be comparable with respect to known covariates.

Both issues are resolved with a core statistical tool: randomization. Unfortunately, health-care studies usually rely on convenience sampling (a nonprobability sampling where the researcher selects whichever individuals are easiest to reach and/or enroll in the study) to recruit participants, as it would be unethical to force someone into a study against his or her will. Thus, randomization occurs after the sample has been gathered, during the allocation of participants to treatment groups (ATLAS used computer software to accomplish this task). The idea behind randomizing participants between treatment groups is that it will balance other factors that may affect the outcome, such as age, gender, and weight (Probstfield, 2012). Canceling out effects from these variables helps to ensure that any difference observed between groups is due exclusively to the treatment imposed by the researcher and not to demographics or other participant characteristics.

## Measuring the Treatment Effect

Tests of statistical significance and their associated p values are common sites in many abstracts and results sections, but one major shortcoming of these calculations is that they are blind to the study design—in essence, they do not tell the whole story. For example, if a treatment group consisted of only women older than age 65 and the control group consisted of only men younger than age 60, it would be impossible to conclude whether the difference in groups was due to age, gender, or the treatment being studied. The researcher in this hypothetical study would still be able to calculate a p value, but that one number would not communicate the major flaws in the design.

Because the ATLAS researchers had a large sample and used a computer for their randomization, their 5- and 10-year tamoxifen groups are nearly identical in all demographic measures (see Table). ATLAS researchers also emphasized that although they reported side effects results on all women in the study, they limited their report on breast cancer outcomes to only those women who were known to be ER positive. The reason for this decision is rooted in the ideas of randomization and generalizability. Notice in the Table that 37% of the women in the study had an unknown ER status. If the researchers had included those women in the breast cancer report, they would have potentially been introducing another source of bias. They would no longer be able to determine whether the two groups had the same proportion of ER-positive patients. The decision they made limits the generalizability of their results to only the ER-positive population, but since that was their initial target population, this was a wise course of action.

**Table 1:**
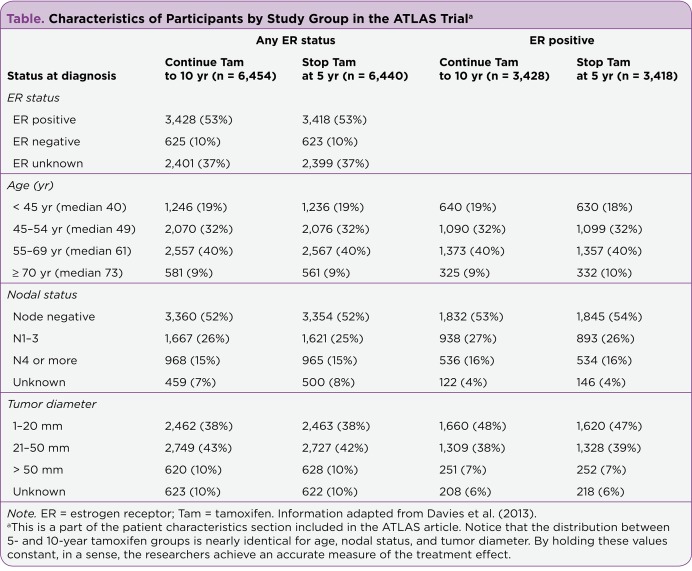
Table 1. Characteristics of Participants by Study Group in the ATLAS Trial

## How Close Is Close Enough?

The ATLAS trial researchers successfully achieved balanced study groups, but how alike do the study groups reasonably have to be to allow an accurate measure of the treatment effect? Again, we turn to statistics. Several measures compare distributions to see whether the differences between them are statistically significant. For quantitative data such as age and weight, a simple student’s t-test or analysis of variance (ANOVA) will suffice (McDonald, 2009). For categorical data such as gender and race, the chi-squared test and Fisher’s exact test are appropriate (McDonald, 2009). Other tests are available, but these are the ones most commonly used.

Normally, researchers are interested in small p values (less than .05) because they suggest that the differences are too great to have occurred through random variation alone. When comparing covariates between treatment groups, however, a significant difference is not desired; this would suggest that the groups are unbalanced. Therefore, large p values (greater than .05) are the target here. However, even a p value greater than .05 does not necessarily guarantee that a specific covariate will not influence the response. One should always consider the p values in the context of sample size as well as prognostic value of the covariate and address these concerns in the discussion section (Scott, 2010).

## Conclusion

In attempting to accurately measure a specific treatment effect, adjusting for covariates that may affect the results is a necessity. It provides for a more reliable measure of the treatment effect while eliminating bias. The best way to account for the effects of these variables is to randomly assign participants to treatment groups and then verify that the groups are statistically alike in these measures.
